# Analysis of using high-precision radiotherapy in the treatment of liver metastases regarding toxicity and survival

**DOI:** 10.1186/s12885-021-08488-y

**Published:** 2021-07-06

**Authors:** Theresa Voglhuber, Kerstin A. Eitz, Markus Oechsner, Marco M. E. Vogel, Stephanie E. Combs

**Affiliations:** 1 Department of Radiation Oncology, University Hospital Klinikum rechts der Isar, Technical University of Munich (TUM), Ismaninger Straße 2, 81675 Munich, Germany; 2 Institute of Radiation Medicine (IRM), Helmholtz Zentrum München, Ingolstädter Landstraße 1, Neuherberg, Germany; 3 Deutsches Konsortium für Translationale Krebsforschung (DKTK), Partner Site Munich, Munich, Germany

**Keywords:** Stereotactic body radiation therapy, Liver metastases, Hepatic metastases, High-precision radiotherapy, Oncology, Outcome, SBRT, Toxicity, Survival

## Abstract

**Background:**

Hepatic metastases occur frequently in the context of many tumor entities. Patients with colorectal carcinoma have already developed liver metastases in 20% at the time of diagnosis, and 25–50% develop metastases in the further course of the disease and therapy. The frequent manifestation and the variable appearance of liver metastases result in an interdisciplinary challenge, regarding treatment management. The aim of this study was to evaluate high-precision stereotactic body radiotherapy (SBRT) for liver metastases.

**Methods:**

A cohort of 115 patients with 150 irradiated liver metastases was analyzed. All metastases were treated between May 2004 and January 2020 using SBRT. A contrast-enhanced computed tomography (CT) was performed in all patients for treatment planning, followed by image-guided high-precision radiotherapy using cone-beam CT. A median cumulative dose of 35 Gy and a median single dose of 7 Gy was applied.

**Results:**

Median OS was 20.4 months and median LC was 35.1 months with a 1-year probability of local failure of 18% (95%-CI: 12.0–24.3%). In this cohort, 18 patients were still alive at the time of evaluation. The median FU-time in total was 11.4 months and for living patients 26.6 months. 70.4% of patients suffered from acute toxicities. There were several cases of grade 1 and 2 toxicities, such as constipation (13.9%), nausea (24.4%), loss of appetite (7.8%), vomiting (10.4%), diarrhea (7.8%), and abdominal pain (16.5%). 10 patients (8.7%) suffered from grade 3 toxicities. Late toxicities affected 42.6% of patients, the majority of these affected the gastrointestinal system.

**Conclusion:**

SBRT is becoming increasingly important in the field of radiation oncology. It has evolved to be a highly effective treatment for primary and metastasized tumors, and offers a semi-curative treatment option also in the case of oligometastatic patients. Overall, it represents a very effective and well-tolerated therapy option to treat hepatic metastases. Based on the results of this work and the studies already available, high-precision radiotherapy should be considered as a valid and promising treatment alternative in the interdisciplinary discussion.

## Background

Due to the rich blood supply, the liver is one of the most common organs that are affected by distant metastases of various primary tumors [1]. In most cases, liver metastases occur in the context of breast cancer, malignant melanoma, lung tumors, colorectal-, ovarian- and pancreatic cancer [2]. For instance, in patients who suffer from colorectal cancer, around 20% of all patients diagnosed have already developed metastases in the liver at the time of diagnosis. 25–50% will develop hepatic metastases in the further course of disease and therapy [3]. The frequent manifestation and the variable appearance of liver metastases result in an interdisciplinary challenge, regarding therapeutical and clinical management [1, 4]. There are many different concepts available for treating liver metastases. Radical surgeries, systemic tumor therapy, radiation therapy, and various ablative procedures such as percutaneous radiofrequency and microwave ablation, transarterial chemoembolization (TACE), cryoablation, and selective internal radioembolization are among the therapeutic options in metastasis therapy. These procedures can also be used in palliative situations and for pain-, as well as for symptom control [5].

Which therapy is best suited for the individual patient depends on many different factors, such as age, general condition, tumor burden, symptoms, image-morphological structure, and extent of the tumor. The therapy decision is therefore usually made in interdisciplinary discussion rounds. Since initial treatments with surgical tumor excision or systemic tumor therapy do not always deliver the best possible treatment result for the patient or many patients are not suitable due to comorbidities and inoperability of the tumor, other options such as stereotactic radiotherapy appear in the field of therapeutical options [1, 4]. Since the introduction of high-precision radiation in the form of stereotactic body radiation therapy (SBRT), fractionated stereotactic radiotherapy (FSRT), stereotactic radiosurgery (SRS), intensity-modulated radiation therapy (IMRT), and volume-modulated arc therapy (VMAT), new technologies have been continuously developed to improve LC and to keep therapy-associated side effects as low as possible by sparing organs at risk [6, 7]. In the past decade, this method has developed into an increasingly popular therapy option. Through the precise, image-based application of high individual doses and the rapid dose-reduction outside the target volume, the surrounding tissue can be best-possibly spared [8]. The high single doses and the hypofractionation lead to better LC, but also involve risks. Late toxicities that have not yet been recorded are possible, especially if the applied dose distribution does not correspond to that of the treatment plan due to various factors such as tumor movements or positioning errors [1, 9]. It has long been known that liver metastases and liver primary tumors react sensitively to radiation, but in recent years radiotherapy has played a less significant role in treatment due to the difficult risk-benefit assessment. Overall, the liver is a very radiation-sensitive organ, which is why the radiation dose must be applied as precisely and locally-confined as possible since otherwise high toxicities and severe liver damage must be expected [1, 10].

Recently, stereotactic irradiation of liver metastases has become a good alternative for patients who cannot be treated surgically, especially those who have oligometastases [1, 4, 10]. Therefore, the purpose of this study was to evaluate the acute and long-term toxicities as well as the oncologic outcome of SBRT for hepatic metastases.

## Methods

### Patients

A cohort of 115 patients with 150 irradiated liver metastases could be acquired for data evaluation. All of the patients were treated between May 2004 and January 2020 at the Department of Radiation Oncology, University Hospital Klinikum rechts der Isar, Technical University of Munich (TUM). Patient characteristics are shown in Table 1. Patients were included in the study who had received SBRT for the treatment of singular and multiple liver metastases. All other applied forms of radiotherapy, as well as whole-liver radiation, were considered as exclusion criteria. Beside, an advanced primary tumor disease, progressive metastases, and a poor general condition of the patient did not constitute a reason for exclusion. For defining SBRT, we used the guidelines of the German DEGRO (Deutsche Gesellschaft für Radioonkologie) working group for stereotactic RT (AG Stereotaxie) [6]. All patients were treated primarily in palliative intention and to prolong progression-free survival. In individual cases, the applied radiotherapy was used as an individual curative attempt. All steps of data acquisition and analysis were approved by the ethics committee of the Medical faculty of TUM (reference number 367/19).

**Table 1:** Patient characteristics

Characteristics	Values
Number of patients (n)	115
Number of LM	150
Gender (n)	
Male	59 (51.3%)
Female	56 (48.7%)
Age at SBRT (median, range) [years]	66.1 (34.7–86.1)
Primary entities	
Rectum	16 (13.9%)
Colon	38 (33.0%)
Colon ascendens	4 (10.5%)
Colon transversum	1 (2.6%)
Colon descendens	2 (5.3%)
Coecum	3 (7.9%)
Left/right flexure	3 (7.9%)
Sigma	16 (42.1%)
Unknown	9 (23.7%)
Esophagus/AEG/stomach	12 (10.4%)
Mamma Ca	20 (17.4%)
NSCLC	5 (4.3%)
Pancreas	4 (3.5%)
Ovary	4 (3.5%)
Others	16 (13.9%)
Symptoms	
Present	8 (7.0%)
Absent	107 (93.0%)
KPS	
100%	6 (4.0%)
90%	83 (55.3%)
80%	46 (30.7%)
≤ 70%	15 (10.0%)
Location of LM	
Left lobe	46 (30.7.4%)
S1	9 (19.6%)
S2	6 (13.0%)
S3	8 (17.4%)
S4	24 (52.2%)
S4a	12 (50.0%)
S4b	4 (16.7%)
Unknown	8 (33.3%)
Right lobe	75 (50.0%)
S5	13 (17.3%)
S6	14 (18.7%)
S7	27 (36.0%)
S8	21 (28.0%)
Overlap	26 (17.3%)
Border S1/S8	1 (3.8%)
Border S2/S3	6 (23.1%)
Border S2/S4a	1 (3.8%)
Border S4a/S4b	2 (7.7%)
Border S4a/S8	6 (23.1%)
Border S5/S6	2 (7.7%)
Border S5/S8	1 (3.8%)
Border S6/S7	2 (7.7%)
Border S7/8	5 (19.2%)
Unknown	3 (2.0%)
Controlled primary	
Yes	103 (89.6%)
No	12 (10.4%)
LM diagnosis	
Synchronous	35 (23.3%)
Metachronous	115 (76.7%)
Metastases in other sites	
Yes	58 (50.4%)
No	57 (49.6%)
Systemic therapy within four weeks before/after RT	
Yes	38 (33.0%)
No	77 (67.0%)
FU-time (median, range) [months]	11.4 (0–123.3)

LM = liver metastasis; SBRT = stereotactic body radiation therapy; AEG = adenocarcinoma of esophagogastric junction; NSCLC = non-small-cell-lung-cancer; KPS = Karnofsky Performing Score; FU = follow-up; synchronous ≤3 months after initial primary diagnosis; metachronous > 3 months after initial primary diagnosis

### Treatment

A contrast-enhanced computed tomography (CT) was performed in all patients with liver metastases for treatment planning. Furthermore, an additional magnetic resonance imaging (MRI) was performed in 98 (65.3%) of the metastases and an additional positron emission tomography-(PET)-CT in 43 (28.7%) metastases to evaluate the tumor volume.

For treatment planning, immobilization was carried out using a vacuum mattress, a wingstep, and an additional knee wedge. Depending on the tumor situation and patient movement, an abdominal press was also used to reduce tumor mobility. Before each treatment session, a cone-beam CT (CBCT) was performed to check the patient’s position in order to ensure high-precision therapy. A median cumulative dose of 35 Gy (range: 12–60 Gy) with a median single dose of 7 Gy (range: 2.5–20 Gy) in 5 (range: 2–16) fractions was applied. Replanning or premature discontinuation of therapy took place in four of the patients. In none of the patients, radiotherapy was the cause of premature discontinuation or rescheduling of the regime.

### Follow-up

Each patient enrolled in the study was simultaneously provided with a detailed plan for follow-up management. All patients were thoroughly examined before, during, and after therapy in order to detect treatment-related side effects as early as possible. The first regular follow-up examination (FU) took place approximately 4–6 weeks after completion of radiotherapy. Each subsequent follow-up was arranged every three months post-therapeutically in the first year, and every 6–12 months thereafter. Depending on the patient’s general condition, tumor progression, and worsening of symptoms, follow-up appointments could also be individualized. Each follow-up appointment included a detailed patient interview with a radiation oncologist, a complete physical examination, and an imaging procedure, in the form of a CT, MRI, PET-CT/MRI, or ultrasound, to evaluate the tumor status. The status of the irradiated liver lesion was assessed in all imaging examinations, regardless of the progression of the primary tumor disease.

Acute and late toxicities were classified according to Common Terminology Criteria Adverse Events (CTCAE) Version 5.0. The adverse events were divided into two groups: acute (< 3 months) and late toxicities (≥ 3 months). Imaging staging examinations and tumor-related symptoms were used to assess tumor growth, as well as local and distant tumor control.

### Statistics

Based on the multiple prevailing competing risks (e.g. death of a patient before the onset of local progression), the probability of local failure was calculated using a competing-risk analysis [11, 12]. This analysis was carried out using R-Statistics (R-Foundation, Vienna). SPSS Statistics Version 25 (IBM, USA) was used for all other statistical analyses. The primary defined endpoints included local tumor control (LC), progression-free survival (PFS), and overall survival (OS) of the patients. Survival analyses were performed using the Kaplan-Meyer method and are reported including the 95% confidence interval (CI). The log-rank test was used to test for significant differences between characteristic values of groups. In patients who were irradiated at multiple liver metastases, the first treatment was used to calculate OS and PFS, for LC, each individual metastasis was evaluated. The LC was calculated from the last day of irradiation until the progress of the treated metastasis or the last known follow-up date, the PFS from the end of treatment until the general tumor progress (excluding the treated metastasis), and OS from the last day of treatment until the death of the patient or the last day the patient was known to be alive. The biologically effective dose with α/β = 10 Gy (BED10) was calculated using the formula BED (Gy) = dose/fraction x fraction number (1 + fraction dose / α/β) [13]. Receiver operating characteristics analysis (ROC) was used to define thresholds for grouped variables (e.g. influence of PTV on LC and OC) and Cox regression for subgroup analysis. All variables for which no suitable threshold could be determined in the ROC analysis were tested as continuous variables (e.g. BED10). A *p*-value < 0.05 was considered significant.

## Results

The median age of patients at initial diagnosis of liver metastases was 65.0 years (range: 34.6–86.1 years) and the median age at initiation of therapy was 66.1 years (range: 34.7–86.1 years). In 58 (58/115, 50.4%) patients, we saw a diffuse multiple tumor spread at the time of therapy planning and we classified 88 (88/115, 76.5%) patients as oligoprogressive. The primary tumor was controlled in 103 (103/115, 89.6%) patients prior to radiotherapy and 57 (57/115, 49.6%) patients suffered from isolated liver metastases and showed no further tumor manifestation. 8 (8/115, 7.0%) patients showed tumor-related symptoms, which were nausea, cholestasis, icterus, abdominal pain, and digestive problems.

In five of these eight symptomatic patients, a clear improvement in symptoms was achieved by the applied radiotherapy. The remaining three persons did not describe a significant improvement of tumor-related symptoms after radiotherapy, but also did not report a worsening of their symptoms. Liver metastases occurred synchronously in 23.3% (35/150) of the cases and metachronously to the diagnosis of the primary tumor in 76.7% (115/150). The median time span between primary tumor diagnosis and the occurrence of liver metastases was 18.8 months (range: 0–257.0 months).

In this cohort, 38 (38/115, 33.0%) patients were systemically treated within 4 weeks before/after radiotherapy and 14 of the patients underwent surgical removal of liver metastases prior to radiotherapy and SBRT was used as local second-line therapy, in case of tumor progression or remaining residual metastasis. The median irradiated planning target volume (PTV) was 116.8 ml (range: 6.2–3707.6 ml). Planning, volume, and treatment parameters are shown in Table 2.

**Table 2:** Radiation parameters and treatment characteristics

	Median	Minimum	Maximum
PTV (ml)	116.8	6.2	3707.6
GTV (ml)	30.5	0.5	691.7
TD (Gy)	35.0	12.0	60.0
SD (Gy)	7.0	2.5	20.0
Fractions	5.0	2.0	16.0
Isodose (%)	60.0	60.0	100.0
PTV-Dmax	56.0	23.1	94.2
PTV-D2%	59.5	24.2	93.3
PTV-D50%	52.3	21.8	73.9
PTV-D98%	39.3	9.8	55.1
GTV-D50%	57.7	23.0	85.3
BED_10_	59.5	19.2	180.0

PTV = planning target volume; GTV = gross tumor volume; TD = total dose; SD = single dose; BED = biological equivalent dose

### Outcome

**Fig. 1:**
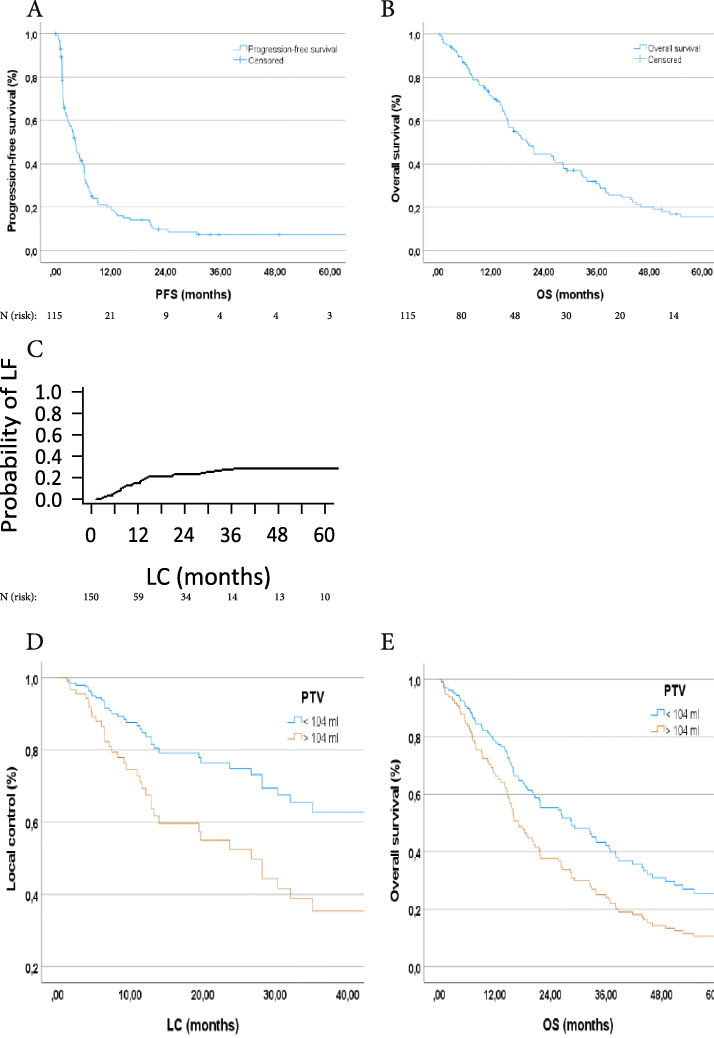
**A** Progression-free survival of patients with LM treated with high-precision radiotherapy; **B** Overall survival of treated patients; **C** Probability of local failure; **D** Local tumor control divided into planning traget volume (PTV) </> 104 ml and adjusted for dose (*p* = 0.040); **E** OS divided into PTV </> 104 ml and adjusted for dose (*p* = 0.036)

In this cohort, 18 (15.7%) of the patients were still alive at the time of evaluation. Median OS in this cohort was 20.4 months (95%-CI: 16.2–24.5 months; Fig. 1 B). OS was 86.9% at 6 months, 71.8% at 12 months, 54.1% at 18 months, and 44.6% at 24 months. The median PFS of the irradiated liver patients was 4.3 months (95%-CI: 3.2–5.4 months; Fig. 1A). After 12 months only 20.1% of the patients did not suffer from distant tumor progression. The analysis of LC also played a major role throughout the evaluation. The median LC in the cohort of patients with irradiated liver metastases was 35.1 months (95%-CI: 0.8–69.4 months). A competing-risk analysis was used to estimate the probability of local recurrence, with a 1-year probability of local failure after radiotherapy of 18% (95%-CI: 12.0–24.3%; Fig. 1C), see also Table 3.

**Table 3:** LF, PFS, and OS in total and depending on time

		LF	PFS	OS
**Event (progress/death) - absolute/ (%)**		41 (27.3)	99 (86.1)	97 (84.3)
**No event - absolute/ (%)**		109 (72.7)	16 (13.9)	18 (15.7)
**Time (months)**		median: 35.1	median: 4.3	median: 20.4
**95%-CI (months)**		0.8–69.4	3.2–5.4	16.2–24.5
	**Proportion surviving after**			
	**6 months**	**12 months**	**18 months**	**24 months**
**OS**	87%	72%	54%	45%
**PFS**	40%	20%	14%	10%
	**Probability of LF after**			
	**6 months**	**12 months**	**18 months**	**24 months**
**LF**	7%	18%	21%	23%

LF = local failure; PFS = progression-free survival; OS = overall survival; CI = confidence interval

Patients with irradiated liver metastases were divided into two groups based on PTV and adjusted by dose using Cox regression. There was a significant difference in OS and LC of patients with smaller tumor volume (OS: *p* = 0.036; LC: *p* = 0.040; Fig. 1 D and E). At the same time, the influence of PTV on OS and LC was also tested as a continuous variable. There also was a significant effect on both, local control (*p* = 0.018) and overall survival (p = < 0.001). We also tested the influence of BED10 on OS and LC as a continuous prognostic variable. No significant influence of BED10 on OS and LC could be detected (OS: *p* = 0.051; LC: *p* = 0.055).

### Treatment toxicity

The median FU time of the total collective was 11.4 months (range: 0–123.3 months) and for the proportion of living patients 26.6 months (range: 1.5–121.5 months). A total of 3 patients were classified as lost-to-follow-up. However, they were still included in the toxicity evaluation, as each of them participated in at least one FU appointment. The most common acute side effects of therapy occurred during or immediately after irradiation. 70.4% (81/115) of the patients suffered from acute toxicities. The majority of these affected the gastrointestinal tract and the digestive system. There were several cases of grade 1 and 2 toxicities, such as constipation (13.9%), nausea (24.4%), loss of appetite (7.8%), vomiting (10.4%), diarrhea (7.8%), and abdominal pain (16.5%). Besides, two patients experienced acute, mild swallowing difficulties, which improved rapidly over the course of the study. A further two patients were diagnosed with acute postradiogenic grade 2 hepatitis via CT, but had no symptoms and did not require further intervention. In 10 of the patients (8.7%), grade 3 adverse events occurred in the time after the applied radiotherapy, see also Table 4.

**Table 4:** Patients with grade 3 toxicity after high-precision radiotherapy of liver metastases

Patients with G3 toxicity (***n*** = 10)	TD (Gy)	SD (Gy)	BED_**10**_ (Gy)
**Esophagusstenosis**	42.0	3.0	54.6
**Subileus**	25.0	5.0	37.5
**Cholestasis due to biliary stenosis**			
**Patient 1**	37.5	12.5	84.4
**Patient 2**	35.0	7.0	59.5
**Patient 3**	35.0	7.0	59.5
**Patient 4**	25.0	5.0	37.5
**Colic type intestinal cramp**			
**Patient 1**	25.0	5.0	37.5
**Patient 2**	35.0	7.0	59.5
**Liver failure/encephalopathy**	35.0	7.0	59.5
**Cholangitis**			
**Patient 1**	25.0	5.0	37.5
**Patient 2 (recurrent irradiation)**	14.0 (25.0)	7.0 (5.0)	23.8 (37.5)
**Liverabscess**	35.0	7.0	59.5

Among them, one patient with esophageal stenosis, one with subileus, and one with postradiogenic liver abscess. The patient with postradiogenic liver abscess was admitted to the emergency department due to recurrent fever attacks with cholestasis. The abscess was revealed through sonography and got surgically repaired, resulting in rapidly decreasing cholestasis parameters. All therapy-associated side effects are shown in Table 5.

**Table 5:** Acute and late toxicities after high-precision radiotherapy of liver metastases

Acute toxicity (***n*** = 115)	Grade 1 absolute/ (%)	Grade 2 absolute/ (%)	Grade 3 absolute/ (%)
**Nausea**	20 (17.4)	8 (7.0)	0 (0.0)
**Vomiting**	7 (6.1)	5 (4.3)	0 (0.0)
**Abdominal pain**	17 (14.8)	2 (1.7)	0 (0.0)
**Loss of weight**	3 (2.6)	1 (0.9)	0 (0.0)
**Loss of appetite**	9 (7.8)	0 (0.0)	0 (0.0)
**Diarrhea**	7 (6.1)	2 (1.7)	0 (0.0)
**Constipation**	15 (13.0)	1 (0.9)	0 (0.0)
**Flatulence**	3 (2.6)	0 (0.0)	0 (0.0)
**Dyspepsia/Reflux**	4 (3.5)	0 (0.0)	0 (0.0)
**Swallowing difficulties**	2 (1.7)	0 (0.0)	0 (0.0)
**Rad. liver-parenchymal abnormalities**	2 (1.7)	0 (0.0)	0 (0.0)
**Radiogenic hepatitis**	0 (0.0)	2 (1.7)	0 (0.0)
**Cholestasis/biliary stenosis**	1 (0.9)	1 (0.9)	0 (0.0)
**Subileus/Corpostasis**	0 (0.0)	0 (0.0)	1 (0.9)
**Esophagusstenosis**	0 (0.0	0 (0.0)	1 (0.9)
**Fatigue**	38 (33.0)	13 (11.3)	0 (0.0)
**Erythema/Radiodermatitis**	1 (0.9)	1 (0.9)	0 (0.0)
**Thoracic−/ribpain**	0 (0.0)	1 (0.9)	0 (0.0)
**Fever/chills/sweating**	6 (5.2)	2 (1.7)	0 (0.0)
**Hyperpigmentation/scarring**	1 (0.9)	0 (0.0)	0 (0.0)
**Dizziness**	1 (0.9)	0 (0.0)	0 (0.0)
**Rad. pneumonitis**	0 (0.0)	2 (1.7)	0 (0.0)
**Late toxicity (n = 115)**	**Grade 1** **absolute/ (%)**	**Grade 2** **absolute/ (%)**	**Grade 3** **absolute/ (%)**
**Nausea**	4 (3.5)	2 (1.7)	0 (0.0)
**Vomiting**	2 (1.7)	2 (1.7)	0 (0.0)
**Abdominal pain**	13 (11.3)	8 (7.0)	2 (1.7)
**Loss of weight**	4 (3.5)	3 (2.6)	0 (0.0)
**Loss of appetite**	5 (4.3)	2 (1.7)	0 (0.0)
**Diarrhea**	6 (5.2)	1 (0.9)	0 (0.0)
**Constipation**	3 (2.6)	3 (2.6)	0 (0.0)
**Flatulence**	2 (1.7)	1 (0.9)	0 (0.0)
**Dyspepsia/Reflux**	1 (0.9)	0 (0.0)	0 (0.0)
**Cholestasis/biliary stenosis**	4 (3.5)	1 (0.9)	4 (3.5)
**Rad. liver-parenchymal abnormalities**	0 (0.0)	2 (1.7)	0 (0.0)
**Cholangitis**	0 (0.0)	1 (0.9)	2 (1.7)
**Liver failure/encephalopathy**	0 (0.0)	0 (0.0)	1 (0.9)
**Liverabscess**	0 (0.0)	0 (0.0)	1 (0.9)
**Fatigue**	8 (7.0)	9 (7.8)	0 (0.0)
**Erythema/Radiodermatitis**	1 (0.9)	0 (0.0)	0 (0.0)
**Thoracic−/ribpain**	1 (0.9)	2 (1.7)	0 (0.0)
**Rad. lung abnormalities**	2 (1.7)	0 (0.0)	0 (0.0)
**Hyperpigmentation/scarring**	2 (1.7)	1 (0.9)	0 (0.0)
**Dry cough**	1 (0.9)	1 (0.9)	0 (0.0)
**Numbness in the irradiation field**	0 (0.0)	1 (0.9)	0 (0.0)

In addition to gastrointestinal toxicities, other acute grade 1–2 adverse events occurred post-therapeutically and included the following symptoms: fatigue (44.3%), fever and chills (6.9%), radiogenic pneumonitis (1.7%), and occasionally skin erythema and post-radiogenic skin abnormalities, such as hyperpigmentation (2.6%). Late toxicities affected 42.6% of this cohort. Gastrointestinal problems were among the most common side effects here as well. Over the long term, 34.8% of patients experienced recurrent or persistent grade 1 and 2 gastrointestinal adverse events, including abdominal pain (18.3%), nausea (5.2%), weight loss (6.1%), constipation (5.2%), loss of appetite (6.1%) and cholestasis (4.3%).

## Discussion

Within the scope of this study, a group of 115 persons with 150 liver metastases was evaluated which received high-precision radiotherapy at the Department of Radiation Oncology, University Hospital Klinikum rechts der Isar, Technical University of Munich (TUM) from May 2004 to January 2020 as part of their treatment plan. This group is one of the largest cohorts analyzed in the literature to date concerning high-precision radiotherapy for liver metastases. We focused on survival parameters and therapy-associated toxicity. Looking at the results for the cohort, 6-, 12-, and 24-month survival rates of 87, 72, and 45% for OS and 7, 18, and 23% for the probability of metastatic recurrence were found. Metastases are a major problem in every oncological disease. The further the tumor spreads and the more organs and structures are affected, the worse the prognosis of the patient and the more limited and difficult the therapy management becomes. If, for example, metastatic penetration of the liver is found, the median survival of the patient is expected to be about 6 months, regardless of whether extrahepatic tumor localization exists [14].

To date, surgical removal of metastases in the liver area has proven to be the gold standard of therapy and the only potentially curative procedure in oligometastatic patients. Especially for isolated metastases, surgery is the therapy of choice. Depending on the organ, tumor spread, and patient’s condition, a distinction is made between open and laparoscopic variants, and between anatomical and non-anatomical resection [15]. However, surgery is a very invasive procedure and is only applicable in very few patients, especially in the case of liver metastases. Due to this, other ablative, local treatment methods are needed to offer an alternative therapy to patients who are not suitable for surgical excision. Various ablation methods, such as radiofrequency-, microwave-, and cryoablation, also play an important role in the treatment of metastases. However, these are also invasive and associated with more serious complications and side effects [5]. Stereotactic hypofractionated high-precision radiotherapy represents a non-invasive alternative therapy option and has developed into a promising method for the treatment of a wide variety of tumors and tumor metastases in recent years. Up to now, SBRT, FSRT and SRS have been widely used for the treatment of lung, prostate, and brain tumors and SBRT is now developing into a promising method also for the treatment of liver metastases [16].

The results of the cohort in this study are generally in good agreement with those of other studies, both in terms of OS and LC. Looking at the published LC rates, a broad range of 50–100% is reported [17–21]. Due to the frequent occurrence of competing events, no LC rate was determined, but the probability of local failure was estimated using a competing-risk analysis. LC of liver metastases after radiotherapy has already been associated with the amount of BED10 applied in some studies. Chang et al. reported an 18-month LC rate of 80% vs. 30% with application of a BED10 from ≥75 Gy and < 75 Gy [22]. Furthermore, Lanciano et al. published a 2-year LC rate of 75% was found with a BED10 of ≥100 Gy and 38% with a BED10 of < 100 Gy [23]. In this cohort of 115 patients, the association between higher BED10 and better LC was borderline significant (*p* = 0.055). In 8 out of 41 (19.5%) post-therapeutic progressive liver metastases, progress was recorded with an applied BED10 of ≥72 Gy. All other progressive liver metastases received BED10 < 72 Gy. Post-interventional toxicities are an important limiting factor for any new therapy. The advantage of hypofractionated stereotactic irradiation is extremely good tolerability. Due to the non-invasive, exact percutaneous application and the recess and thus sparing of surrounding healthy tissue, the therapy-associated side effects are very mild and easily treatable in most cases. Overall, the irradiation of liver metastases is very well tolerated [17–21]. The side effects are not very pronounced and CTCAE grade 3–4 toxicities occur in 1–10% of cases only [1]. Since the liver is a very radiation-sensitive organ, it is of great importance to preserve the surrounding healthy structures and to protect them from unnecessary radiation exposure, as this is associated with an increased risk of toxicities. In the past, radiation-induced liver disease (RILD) has played a major role as a side effect of conventional radiotherapy regimens, with patients having pre-existing liver dysfunction often being at the highest risk. In the case of liver metastases, the risk of developing RILD after radiotherapy is less than 1% according to the published results [1, 10].

Several different studies are also devoted to side effects in the bile ducts. According to Osmundson et al., the most common grade 3 toxicities are hepatobiliary stenoses and bile stasis. In their cohort, the effect of SBRT was investigated by irradiating various liver primary tumors and metastases. The results showed that, although grade 3 adverse events occurred in 18.8% of cases, irradiation of liver metastases was best tolerated and the least number of treatment-related complications occurred [24]. In the analyzed cohort of this study, we also found various hepatobiliary toxicities. In 6.1%, mild cholestasis was observed, in 3.5% a grade 3 biliary stenosis, in 1.7% a postradiogenic hepatitis, in 0.9% recurrent grade 2 cholangitis, and in 1.7% a grade 3 cholangitis. Both bile duct and gallbladder toxicities are considered to be rare after stereotactic radiotherapy. It is unclear which fractionation scheme is considered safe to prevent side effects in this area, but Eriguchi et al. declared a dose of 40 Gy in 5 fractions as safe for tumors in the liver hilum [25]. Similarly, the pathogenesis of stenosis of the bile ducts after radiotherapy is not yet fully understood. It is assumed that radiation-induced fibrosis causes occlusion or that interaction of systemic therapy, surgery and radiotherapy has an occlusive effect. In rare cases, gallbladder toxicities are described in the context of radioembolization with Yttrium-90, but not in SBRT [25]. This is also supported by the results of this study since no patient has experienced cholecystitis, gallbladder rupture, or other side effects. Other organs at risk near the liver are the stomach, the small and large intestine. These are the organs most frequently affected by therapy-associated side effects in every SBRT. The severity can range from mild nausea to severe gastrointestinal hemorrhages and perforations. In recent years, there have been isolated reports of patients with hemorrhagic gastritis and duodenal ulcers following radiation; however, as there are no uniform dose restrictions for intestinal organs at risk, extreme caution is advised when outlining the radiation plan. According to some studies, a cumulative dose of 30 Gy and a single dose of 10 Gy for duodenum and colon should not be exceeded to prevent severe toxicity [26, 27]. Further side effects of the therapy concern the skin in the irradiated area and the thoracic wall. SBRT of lung malignancies and breast cancer have already been reported to cause severe chest pain and pathological rib fractures [28]. In the group of patients in this study no pathologic fracture occurred in any of the patients, but there were isolated reports of chest and rib pain. Although the esophagus is one of the less affected organs at risk, there are also some cases of radiogenic side effects. According to Stephans et al., the occurrence of toxicities correlates with the application of a cumulative dose of more than 50 Gy while receiving systemic tumor therapy, often with VEGF-modulating drugs [29]. In this cohort, esophageal toxicities occurred in three patients. Two of them reported mild swallowing difficulties that did not require further treatment, but one patient developed grade 3 esophageal stenosis after stereotactic treatment. One of the other more serious complications following radiotherapy was a patient who developed a liver abscess in the radiation field. There is little information on this type of SBRT toxicity and there are almost no publications on this subject, as liver abscesses are more likely to occur during chemoembolization or radiofrequency ablation rather than SBRT [30, 31]. Nevertheless, there are a few isolated case reports that aim to draw attention to this side effect of radiotherapy, which should not be underestimated. In the cohort of Mahadevan et al. a grade 3 liver abscess occurred in one patient during SBRT of liver cholangiocellular carcinomas, and Macomber et al. also showed two cases of an abscess after liver radiation [32, 33]. The irradiated patient with liver abscess in the cohort of this study was rapidly discharged after surgical repair and systemic antibiotic treatment. The analysis and the available results indicate that stereotactic high-precision radiotherapy is a versatile, promising and above all, well-tolerable therapy option. However, there are some limitations of the study. Among them are the retrospective design of the analysis and the short follow-up period. Furthermore, the very heterogeneously applied irradiation regimes are also among the limiting factors, as there are no uniform recommendations regarding dose and fractionation. For this reason, it is advisable to continue to conduct larger-scale studies in this area to fully exploit the potential of this therapeutic method.

This work aimed to obtain a clearer picture of the therapeutic option of stereotactic high-precision irradiation and its field of application, as well as to evaluate the advantages and disadvantages for the patient in the best possible way.

## Conclusion

Stereotactic hypofractionated high-precision radiotherapy is becoming increasingly important in the field of radiooncology. Overall, it represents a very effective and well-tolerated therapy option. The low number of serious side effects is due to the image-guided application of the radiation and the rapid dose reduction outside the target volume, as the surrounding healthy tissue is spared as much as possible. In particular, patients with oligometastasis and oligoprogression benefit from this method, as do patients for whom other, more invasive measures are not suitable due to comorbidities. However, clear guidelines and recommendations are needed. The results of this analysis are helpful to perform an individualized risk-benefit analysis of each patient with liver metastases and thus to create clearer structures in the treatment strategy of these patients. Based on the results of this work and the studies already available, stereotactic radiotherapy for metastases of the liver should be considered as a valid and promising treatment alternative in the interdisciplinary discussion, alongside systemic chemo-, immuno-, and hormone therapy. Nevertheless, further prospective studies are indispensable for the assessment of the clinical benefit over the long term.

## Data Availability

All datasets used to create and support the results and conclusions of this article can be found within the article.

## Abbreviations

SBRT: stereotactic body radiation therapy
FSRT: fractionated stereotactic radiotherapy
SRS: stereotactic radiosurgery
IMRT: intensity-modulated radiation therapy
VMAT: volume-modulated arc therapy
AEG: adenocarcinoma of esophagogastric junction
GOT: glutamate oxaloacetate transaminase
GPT: glutamate pyruvate transaminase
LDH: lactate dehydrogenase
TACE: transarterial chemoembolization
LC: local control
LF: local failure
PFS: progression-free survival
OS: overall survival
CBCT: cone-beam-CT
CT: computed tomography
MRI: magnet resonance imaging
PET-CT/MRI: positron emission tomography-CT/MRI
BED: biological equivalent dose
DEGRO: Deutsche Gesellschaft für Radioonkologie
LM: liver metastasis
RT: radiotherapy
NSCLC: non-small-cell-lung-cancer
KPS: Karnofsky Performing Score
FU: follow-up
CTCAE: Common Terminology Criteria Adverse Events
PTV: planning target volume
GTV: gross tumor volume
TD: total dose
SD: single dose
CI: confidence interval
ROC: receiver operating characteristics
